# Supplementary material for: High fat diet induces microbiota-dependent silencing of enteroendocrine cells
Source: eLife. 2019 Dec 3;8:e48479. doi: 10.7554/eLife.48479 (PMC6937151; doi:10.7554/eLife.48479)
Supplement: Transparent reporting form [file elife-48479-transrepform.docx]

***eLife’s* transparent reporting form**

We encourage authors to provide detailed information *within their submission* to facilitate the interpretation and replication of experiments. Authors can upload supporting documentation to indicate the use of appropriate reporting guidelines for health-related research (see [EQUATOR Network](http://www.equator-network.org/%20)), life science research (see the [BioSharing Information Resource](https://biosharing.org/)), or the [ARRIVE guidelines](http://www.plosbiology.org/article/info:doi/10.1371/journal.pbio.1000412) for reporting work involving animal research. Where applicable, authors should refer to any relevant reporting standards documents in this form.

If you have any questions, please consult our Journal Policies and/or contact us: [editorial@elifesciences.org](mailto:editorial@elifesciences.org).

**Sample-size estimation**

- You should state whether an appropriate sample size was computed when the study was being deacesigned
- You should state the statistical method of sample size computation and any required assumptions
- If no explicit power analysis was used, you should describe how you decided what sample (replicate) size (number) to use

Please outline where this information can be found within the submission (e.g., sections or figure legends), or explain why this information doesn’t apply to your submission:

-The sample size for each experiment is indicated in each figure legend. For most figures, the sample size can also be directly visualized as the data points in each graph is represented as individual solid dots.

-The statistic for sample size determination can be found in the Methods section.

**Replicates**

- You should report how often each experiment was performed
- You should include a definition of biological versus technical replication
- The data obtained should be provided and sufficient information should be provided to indicate the number of independent biological and/or technical replicates
- If you encountered any outliers, you should describe how these were handled
- Criteria for exclusion/inclusion of data should be clearly stated
- High-throughput sequence data should be uploaded before submission, with a private link for reviewers provided (these are available from both GEO and ArrayExpress)

Please outline where this information can be found within the submission (e.g., sections or figure legends), or explain why this information doesn’t apply to your submission:

-The replicate information can be found in each figure legend and the Methods section.

- All the data presented in this paper are shown as biological replicates defined as individual fish (for all imaging data) or as pooled fish samples (for all mRNA data).

- We did not exclude outliers in our data analysis.

-The 16S ribosomal gene sequencing data was deposited at SRA under Bioproject accession number PRJNA532723. This information is available in the Methods section.

**Statistical reporting**

- Statistical analysis methods should be described and justified
- Raw data should be presented in figures whenever informative to do so (typically when N per group is less than 10)
- For each experiment, you should identify the statistical tests used, exact values of N, definitions of center, methods of multiple test correction, and dispersion and precision measures (e.g., mean, median, SD, SEM, confidence intervals; and, for the major substantive results, a measure of effect size (e.g., Pearson's r, Cohen's d)
- Report exact p-values wherever possible alongside the summary statistics and 95% confidence intervals. These should be reported for all key questions and not only when the p-value is less than 0.05.

Please outline where this information can be found within the submission (e.g., sections or figure legends), or explain why this information doesn’t apply to your submission:

- Statistical reporting for raw data, statistical tests and p value can be found in each figure legend and method section.

- All statistical analyses are also posted in the DataDryad submission associated with this article.

(For large datasets, or papers with a very large number of statistical tests, you may upload a single table file with tests, Ns, etc., with reference to sections in the manuscript.)

**Group allocation**

- Indicate how samples were allocated into experimental groups (in the case of clinical studies, please specify allocation to treatment method); if randomization was used, please also state if restricted randomization was applied
- Indicate if masking was used during group allocation, data collection and/or data analysis

Please outline where this information can be found within the submission (e.g., sections or figure legends), or explain why this information doesn’t apply to your submission:

- For all experiments, embryos from wildtype or transgenic fish were randomly assigned to test groups prior to the treatment. No specific method was used for randomizing.

- This information can be found in the Methods section.

**Additional data files (“source data”)**

- We encourage you to upload relevant additional data files, such as numerical data that are represented as a graph in a figure, or as a summary table
- Where provided, these should be in the most useful format, and they can be uploaded as “Source data” files linked to a main figure or table
- Include model definition files including the full list of parameters used
- Include code used for data analysis (e.g., R, MatLab)
- Avoid stating that data files are “available upon request”

Please indicate the figures or tables for which source data files have been provided:

Source data is provided for the following figures in the DataDryad submission associated with this manuscript:

- Figure 1 I, J

- Figure 2 D, E, F, J

- Figure 3 C, D, J

- Figure 4 E

- Figure 5 A-C, H, I, O-R

- Figure 6 E, F, N-P

- Figure 7 G-K

- Figure 8 A, D, E, F, H

- Supplemental figure 3 D
